# Quantification of localised vascular wedge‐shaped defects in glaucoma

**DOI:** 10.1111/ceo.14134

**Published:** 2022-07-26

**Authors:** Danit Saks, Angela Schulz, Samran Sheriff, Ting Shen, Vivek Gupta, Ayub Qassim, Bronwyn Ridge, Ryan Pham, Jamie Craig, Stuart Graham, the PROGRESSA Study Group

**Affiliations:** ^1^ Department of Clinical Medicine Macquarie University Sydney New South Wales Australia; ^2^ Department of Ophthalmology, Flinders Health and Medical Research Institute Flinders University Adelaide South Australia Australia

**Keywords:** glaucoma, imaging, optical coherence tomography angiography, vascular change

## Abstract

**Background:**

Vascular dysfunction plays a considerable role in glaucoma pathogenesis. Previous glaucoma case studies described localised wedge‐shaped vascular defects, similar to retinal nerve fibre layer (RNFL) wedge defects. This study investigates the prevalence and quantification of this vessel loss, in relation to primary open angle glaucoma (POAG) parameters.

**Methods:**

This study included 608 eyes (351 participants): 192 PROGRESSA study participants (342 eyes) with suspect, preperimetric or early manifest POAG, observed for vascular wedge defect presence (cohort one); an additional 114 individuals (cohort two—208 eyes) with POAG at various stages of progression for wedge characterisation; and 38 controls (56 eyes).

Vascular wedge defects were observed using optical coherence tomography angiography (OCTA). Wedge parameters and vessel densities were quantified using ImageJ software. RNFL and ganglion cell layer inner plexiform layer (GCLIPL) from OCT scans, and mean deviation (Humphrey visual field 24–2) were also assessed.

**Results:**

Vascular wedge defects were found in 45/342 eyes (13.2%) in cohort one, in 41/208 eyes (19.7%) in cohort two and were not found in controls. Wedge defects were mostly inferotemporal (80%), and present at all disease stages. They were associated with visual field loss in the opposite hemisphere, thinner RNFL (*p* < 0.001), thinner GCLIPL (*p* = 0.003), and focal RNFL loss corresponding with the vascular defect region.

**Conclusion:**

Vascular wedge defects are present at all POAG stages even before functional change and are strongly concordant with focal RNFL loss. Further research is needed to explore these defects in particular their temporal relationship with clinical measures of POAG.

## 1 INTRODUCTION

Glaucoma pathophysiology is complex and multifactorial, not limited to elevated intraocular pressure (IOP) as previously thought.^1^, ^2^, ^3^, ^4^ Glaucoma is defined by the gradual loss of ganglion cell axons leading to diffuse or localised retinal nerve fibre layer (RNFL) thinning. Localised RNFL thinning of the inferior and/or superior arcuate bundles often appears as wedge‐shaped defects and often becomes more pronounced as the disease progresses.^5^, ^6^

Previous studies have commented on localised vessel density reductions around the nerve head and peripapillary which correspond with RNFL bundle defects in primary open angle glaucoma (POAG).^7^, ^8^, ^9^, ^10^, ^11^ These vascular wedge defects are visible with *en face* optical coherence tomography angiography (OCTA) imaging. OCTA is a non‐invasive imaging technique which contrasts stable light intensity with the dynamic motion of blood flow, to enable visualisation of the retinal microvasculature.^12^

OCTA imaging gives insight into retinal vasculature at varying depths. Reductions in vessel density have been linked to glaucoma severity^13^ and faster RNFL thinning,^14^ however the most affected vascular level and the most appropriate analysis technique are still to be established.^15^ The distribution and pattern of vascular dropout may also reflect particular glaucoma phenotypes.

To the best of the authors' knowledge, there have been no previous studies which have investigated the extent of vascular wedge defects in glaucoma by identifying and quantifying vascular wedges. This study aims to document their prevalence using participants from a prospective POAG study (PROGRESSA), and also to quantify these vascular defects in terms of size and location, and their relationship to other measurable glaucomatous parameters.

## 2 METHODS

This study was approved by the Macquarie University Human Research Ethics Committee and Southern Adelaide Clinical Research Ethics Committee. Written informed consent was obtained from all participants and all study procedures adhered to the tenets of the Declaration of Helsinki.

### 2.1 Participants

Part one of this study is an investigation into the prevalence of vascular wedge defects in 192 individuals (342 eyes) with suspect, preperimetric or early manifest POAG, enrolled in the Predicting Risk Of Glaucoma: RElevant SNPs with Strong Association (PROGRESSA) study—a longitudinal Australian multicentre study (cohort one).

Part two investigates the characteristics of vascular wedges in an additional 114 individuals (208 eyes—cohort two) with a wider range of glaucoma severity including suspects, preperimetric, early manifest, manifest and advanced POAG. These subjects were recruited from multiple glaucoma specialty clinics, as well as 38 healthy controls (56 eyes), recruited from the Macquarie University Hospital Ophthalmology Clinic. Seven control participants were excluded (aged 56 or younger) in order to age‐match with cohort two. Subjects from cohort one could not be included in the characteristics analysis with cohort two, to ensure that quantification assessments were limited to one type of OCT system (Spectralis).

### 2.2 Inclusion/exclusion criteria

All participants were aged 18 and older. As per the PROGRESSA protocol, participants were diagnosed as glaucoma suspects (normal visual field (VF) with optic nerve head (ONH) or RNFL characteristic of borderline glaucomatous damage with/without glaucoma family history) or ocular hypertensive (IOP ≥22 mmHg on two or more occasions), preperimetric glaucoma (abnormal disc with normal VF), or early manifest glaucoma (abnormal disc with Stage 1 VF defect).

To increase the number of participants with more advanced disease, the second cohort that included manifest POAG cases was also assessed. Participants were excluded if they had narrow iridocorneal angle; steroid‐induced ocular hypertension; retinal, optic nerve or neurological disease causing non‐glaucomatous VF defect or obvious non‐glaucomatous disruption to OCT/OCTA images; inability to obtain disc photography or reliable VF tests.

Control participants had no history of glaucoma or any other ophthalmic conditions that could affect the retina. Treated hypertension was not an exclusion for any group.

### 2.3 Clinical examination

All participants underwent detailed clinical examination including medical history and medications, IOP (applanation tonometry) at visit, visual acuity (Snellen chart), central corneal thickness (ultrasound corneal pachymetry) and retinal imaging (AngioPlex, Carl Zeiss Meditec for cohort one, and Spectralis, Heidelberg OCTA and OCT for cohort two). History of disc haemorrhage (DH) was determined by reviewing the participant's clinical notes up to and including the time of OCTA acquisition. Eyes with ≥1.5 years and at least three visits documented in follow‐up were assessed for DH history.

Subjects in both cohorts had previously undergone VF testing on at least three occasions (Humphrey 24–2 automated perimetry). For inclusion in analysis, tests were considered reliable if there were ≤ 20% fixation losses, ≤20% false negatives and ≤ 25% false positives. The definition of an abnormal visual field was according the HPA staging criteria,^16^ this requires glaucoma hemifield test (GHT) outside normal limits and/or a cluster of three points p < 5% with at least one point p < 1% on the pattern deviation plot. For the purposes of defining the distribution of a focal VF defect, we applied the latter condition of clustered points, to categorise into nasal steps, arcuates, paracentral defects, hemifields and nonspecific loss.

Cohort two also underwent fundus imaging and autorefraction (Topcon; spherical equivalent was calculated as the spherical power + ½ cylindrical power). Control subjects underwent clinical examination including IOP, anterior segment and optic disc, but did not have VF performed or DH assessment.

### 2.4 OCTA acquisition and processing

Cohort one underwent AngioPlex, Zeiss spectral domain OCTA imaging. AngioPlex images were acquired at 6 × 6 mm and were included if the signal strength was ≥7. Automatic segmentation was used to obtain superficial [inner limiting membrane (ILM) + 0, inner plexiform layer (IPL) + 0] and deep [IPL + 0, outer plexiform layer (OPL) + 0] macular and optic disc scans which were then manually overlayed, scan position aligned by matching major structures including vessels, and scans amalgamated to create a singular image file.

Cohort two and controls underwent Spectralis, Heidelberg spectral‐domain OCTA imaging. Macula images were acquired at 8.7 × 4.4 mm, automatically aligned with the fovea, contrast auto set (1:4 for superficial and 1:2 for deep scans) and projection artefact removal turned on. Macular superficial vascular complex (SVC; ILM, IPL‐) and deep capillary plexus (DCP; IPL+, OPL) images were included if OCTA quality was ≥27. Spectralis SVC and DCP were chosen as they best match AngioPlex layer borders.

Scans were assessed independently by two trained assessors (AS and DS) and poor‐quality images or those with artefacts, such as large floaters or multiple prominent scan lines, within the analysis area were excluded. Since Spectralis does not provide automated density values, vessel density and vascular feature analyses were performed using ImageJ software (National Institutes of Health, available at https://imagej.net/Fiji/Downloads) with a method adapted from Elfarnawany et al.^17^ which binarizes the image and quantifies the proportion of vessels within the chosen region. Analysis area was chosen to be 4.4 mm × 4.4 mm as this encompasses the largest square area across both devices. All vessels within this region were included in the analysis.

### 2.5 Wedge defect analysis

**FIGURE 1:**
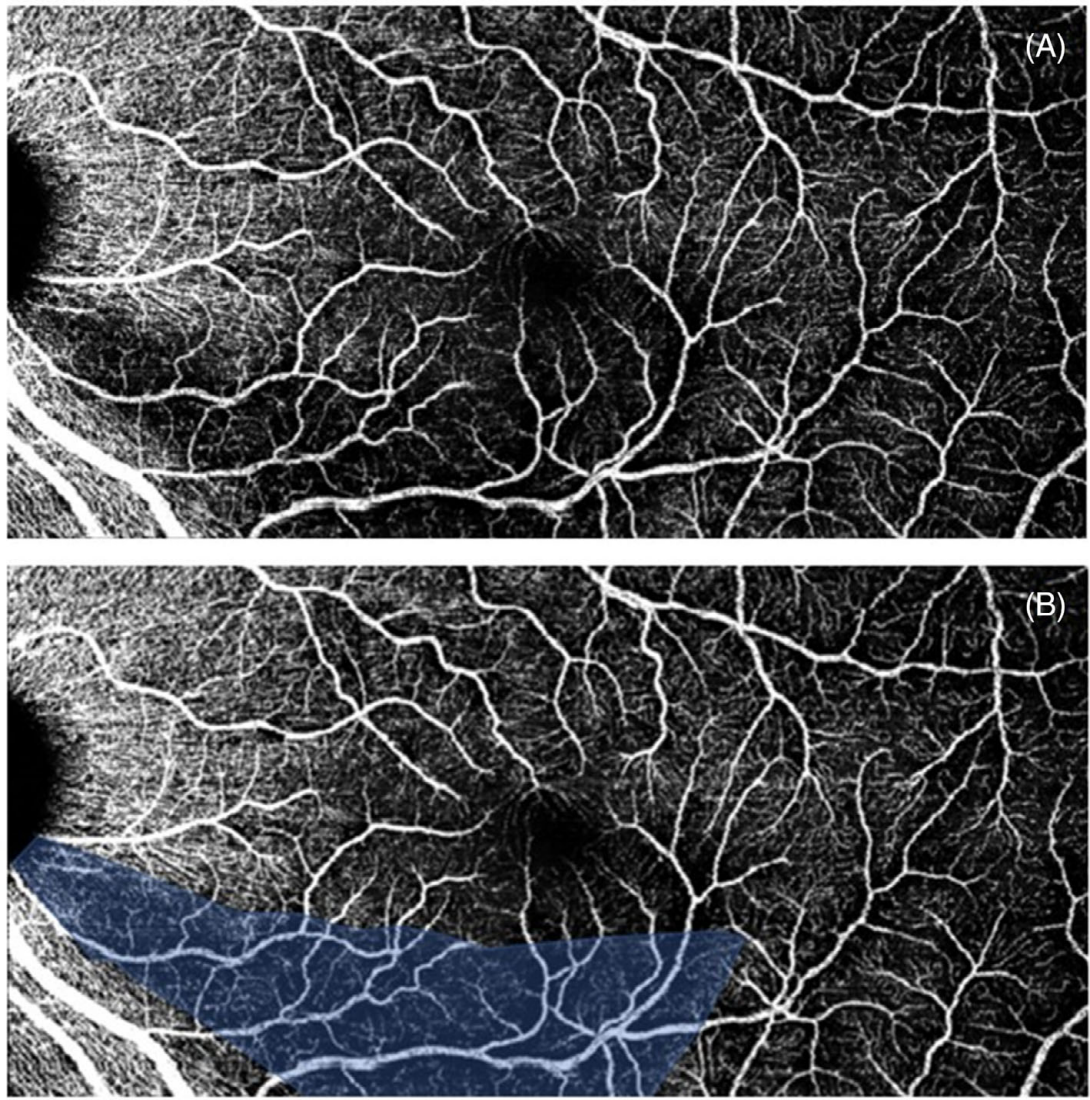
An example of a vascular wedge defect. (A) Indicates the raw export from the Spectralis optical coherence tomography angiography (OCTA) system and (B) indicates the vascular wedge, outlined in ImageJ software. This overlay is used to calculate area of the wedge and for demonstration purposes. Image is binarized to calculate vessel density within the shaded area

Vascular wedge defect presence was confirmed by two masked investigators (AS and DS) by scrutiny of macula and ONH OCTA scans. Wedge outline was manually traced on the macula SVC scans by both observers (wedge area for each eye was outlined by AS and DS, and then the outlined shapes were compared, with an interobserver agreement of 92%) and quantification was finalised in ImageJ software using vessel density (%) as indication of wedge border (Figure 1). The image was binarized and wedge area (mm^2^) and vessel density within the wedge were recorded. Eyes were excluded when the boundary of the wedge closest to the fovea was cropped by the scan area.

### 2.6 OCT acquisition and processing

Peripapillary and macula OCT scans were acquired in cohort two and controls only. Spectralis peripapillary RNFL images were acquired using the RNFL 12° circle scan ~3.4 mm diameter, and macular ganglion cell layer inner plexiform layer (GCLIPL) imaging was acquired using the posterior pole function, which captures 61 B‐scans centred on the fovea. The anatomic positioning system feature was turned on which automatically orients scan alignment with the fovea. Automatic segmentation of the RNFL, GCL and IPL were manually reviewed and corrected for major errors by trained operators (AS and DS) within the Spectralis system. RNFL thicknesses were obtained from the peripapillary RNFLT Classification chart. GCLIPL thickness was recorded using the macular GCL and IPL classification charts, obtained using the deviation map function, by adding the GCL and IPL default slabs thicknesses together. In accordance with Heidelberg guidelines, scans with quality scores >15 were considered acceptable.^18^

### 2.7 Statistical analysis

Statistical analysis was completed using the Statistical Package for Social Sciences (SPSS; Windows, version 25). All analyses were two‐tailed with significance at alpha = 0.05. Descriptive statistics were calculated as mean and standard deviation. Shapiro–Wilk tests revealed non‐normal distribution for all continuous variables, as such non‐parametric tests were used. Demographic characteristics were analysed by Mann–Whitney *U* tests (continuous variables) and Chi‐square tests (categorical). A generalised estimating equations (GEE) model was used for analysis of outcomes. The use of multiple eyes from participants was controlled for and the model was consistently adjusted for sex (factor), and age and OCTA scan quality (covariates).

## 3 RESULTS

### 3.1 Cohort one: Prevalence of vascular wedge defects

Of the 342 eyes in cohort one, 189 were diagnosed as glaucoma suspects, 18 were preperimetric, 90 had early manifest POAG, and 45 were ocular hypertensive. Within this cohort, there were 45 eyes found to have vascular wedge defects (13.2%), which were identified in each diagnostic group, that is, eight suspect eyes, four preperimetric, 32 early manifest POAG and one ocular hypertensive. The relevant demographic and ocular characteristics of these participants are summarised in Table 1.

**TABLE 1:** Demographic and ocular characteristics of cohort one

Characteristics	Cohort one participants (*n* = 342 eyes)	With wedge defects (*n* = 45 eyes)	Without wedge defects (*n* = 297 eyes)	*p* (with vs. without wedge)
Age, years	66.87 (11.28)	72.20 (9.43)	66.06 (11.33)	<0.001*
Sex, number female (%)	188 (55)	30 (67)	158 (53)	0.091
Intraocular pressure (mmHg)	16.12 (3.82)	14.17 (3.15)	16.41 (3.83)	<0.001*
Vertical cup/disc ratio	0.66 (0.12)	0.75 (0.13)	0.64 (0.12)	<0.001*
Central corneal thickness (μm)	548.15 (36.13)	536.78 (30.19)	549.90 (36.70)	0.005*
Best corrected visual acuity decimal equivalent	0.88 (0.19)	0.80 (0.14)	0.90 (0.20)	0.002*
History of disc haemorrhage^a^	26 (8)	18 (40)	8 (3)	<0.001*
Self‐reported family history of glaucoma, number of positive reports (%)^b^	162 (47)	21 (47)	141 (48)	0.783
Glaucoma intervention (%)	116 (34)	28 (62)	88 (30)	<0.001*
Self‐reported history of hypertension (%)	145 (42)	18 (40)	127 (43)	0.700
Self‐reported history of diabetes (%)	65 (19)	6 (13)	59 (20)	0.298

*Note*: Demographic and ocular characteristics for cohort one. Data are mean (*SD*) unless otherwise specified. Continuous variable *p*‐values calculated by Mann–Whitney *U* tests, nominal values by Chi‐square tests.

* *P* = 0.05.

^a^ 37 eyes missing data.

^b^ Data unavailable for 21 eyes.

Eyes with vascular wedge defects compared with those without were statistically more likely to be older, have lower IOP, higher vertical cup/disc ratio, lower central corneal thickness, worse visual acuity and were more likely to have a history of DH (Table 1). Those with wedge defects were also more likely to be currently receiving/have recently received glaucoma intervention including medications or surgeries (selective laser trabeculoplasty, trabeculectomy). There was no difference in the prevalence of hypertension or diabetes between those with and without vascular wedge defects. OCTA scans had a signal strength range of 7 to 10, with a mean of 9.19.

### 3.2 Cohort two and controls: Characteristics of vascular wedge defects

Of the 208 eyes in cohort two, 17 were diagnosed as glaucoma suspects, 98 had preperimetric glaucoma, 74 had manifest POAG and 19 were ocular hypertensive. There were 41 eyes found to have vascular wedge defects (19.7%), which were present in 11 preperimetric glaucoma eyes and 30 manifest POAG eyes. There no wedge defects found in the glaucoma suspects, ocular hypertensives or the control cohort. The relevant demographic and ocular characteristics of cohort two and controls are summarised in Table 2.

**TABLE 2:** Demographic and ocular characteristics of cohort two and controls

Characteristics	Cohort two (*n* = 208 eyes)	Controls (*n* = 56 eyes)	*p* (controls vs. glaucoma eyes)
Age, years	67.22 (8.64)	66.18 (6.22)	0.070
Sex, number female (%)	127 (61)	27 (43)	0.015*
Intraocular pressure (mmHg)	13.49 (2.42)	15.15 (4.06)	0.001*
Vertical cup/disc ratio	0.67 (0.13)	0.51 (0.09)	<0.001*
Central corneal thickness (μm)	542.17 (37.26)	547.13 (32.80)	0.051
Best corrected visual acuity decimal equivalent	0.96 (0.14)	0.93 (0.12)	0.064
Self‐reported family history of glaucoma, number of positive reports (%)	123 (60)	4 (7)	<0.001*
Glaucoma intervention (%)	194 (93)	‐	‐
Self‐reported history of hypertension (%)	84 (40)	18 (32)	0.424
Self‐reported history of diabetes (%)	14 (7)	1 (2)	0.142

*Note*: Demographic and ocular characteristics for cohort two and controls. Data are mean (*SD*) unless otherwise specified. Continuous variable *p*‐values calculated by Mann–Whitney *U* tests, nominal values by Chi‐square tests.

Compared with controls, cohort two were statistically more likely to be female, with lower IOP, higher cup/disc ratio and lower corneal thickness (Table 2). OCTA scans had a quality score, obtained from the system, with a range of 28 to 44, and a mean of 35.94.

Within cohort two, eyes with vascular wedge defects and without vascular wedge defects were compared for their demographic and ocular characteristics (Table 3).

**TABLE 3:** Demographic and ocular characteristic comparison of eyes with wedge defects and eyes without wedge defects

Characteristics	With wedge defects (*n* = 41 eyes)	Without wedge defects (*n* = 167 eyes)	*p* (with vs. without wedge)
Age, years	66.46 (8.19)	67.41 (8.76)	0.507
Sex, number female (%)	26 (63)	101 (61)	0.730
Intraocular pressure (mmHg)	13.71 (2.85)	13.44 (2.31)	0.844
Vertical cup/disc ratio	0.74 (0.11)	0.66 (0.13)	<0.001*
Central corneal thickness (μm)	555.69 (44.67)	539.24 (34.92)	0.069
Best corrected visual acuity decimal equivalent	0.95 (0.14)	0.96 (0.14)	0.497
History of disc haemorrhage^a^	11 (26)	5 (3)	<0.001*
Self‐reported family history of glaucoma, number of positive reports (%)	23 (56)	100 (60)	0.659
Glaucoma intervention (%)	35 (85)	159 (95)	0.071
Self‐reported history of hypertension (%)	12 (29)	72 (43)	0.129
Self‐reported history of diabetes (%)	2 (5)	12 (7)	0.482

*Note*: Demographic and ocular characteristics of eyes with vascular wedge defects compared with those without. Data are mean (*SD*) unless otherwise specified. Continuous variable *p*‐values calculated by Mann–Whitney *U* tests, nominal values by Chi‐square tests.

^a^ Nine eyes missing data.

Eyes with wedge defects compared with those without, had higher vertical cup/disc ratios and were more likely to have a history of DH (Table 3).

Vascular wedge defects were most observed in the inferotemporal region (33 eyes, 80.5%), including six eyes which had both inferior and superior wedge defects. In eyes with more than one defect, the most prominent wedge, as determined by size and dark intensity (confirmed by both observers, AS and DS), was selected for further analysis. Thirty‐three wedge defects were able to be quantified, due to the location of the wedge relative to the standardised scan area. Wedge defect area was significantly varied across the cohort ranging from 0.43 to 17.75 mm^2^ (mean = 9.29 ± 4.53 mm^2^). Vessel density within the wedge defect ranged from 6.47% to 20.87% (mean = 12.18 ± 3.80%).

**FIGURE 2:**
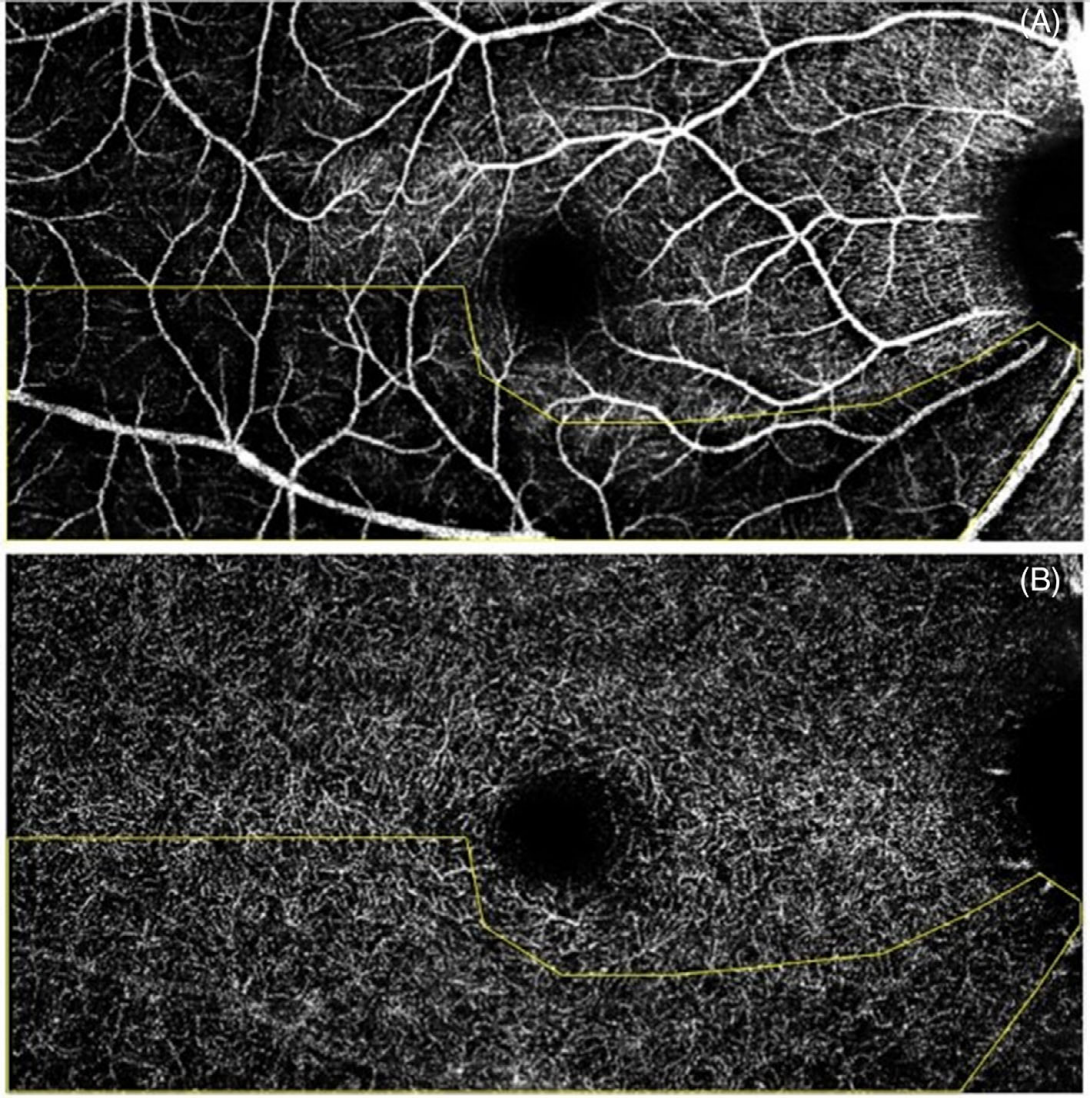
Example of a vascular wedge defect at the superficial vascular complex (top image) with overlay indicating the arcuate trajectory of the wedge and its corresponding deep capillary plexus scan (bottom image)

Vascular wedge defects are identifiable at the SVC (Figure 2), with sometimes minor visible vessel loss near the ONH at the DCP, not enough to identity defects. Global macular SVC and DCP densities were not significantly different between those with and without wedge defects (*p* = 0.149 and *p* = 0.746, respectively) in a GEE model adjusted for age, sex and image quality (Table 4). Vessel density at the SVC level was significantly reduced between glaucoma and control eyes (*p* = 0.001) yet not at the DCP (*p* = 0.253). Vessel density at the DCP within the wedge region (overlayed from SVC) was found to be 79% of the global macula DCP density. Image quality was significantly correlated with both SVC and DCP densities (both *p* < 0.001 and Spearman's *r* = 0.5).

**TABLE 4:** Global macular vessel density at the superficial and deep vascular levels

Vessel density	Cohort two (*n* = 208 eyes)	With wedge defects (*n* = 41 eyes)	Without wedge defects (*n* = 167 eyes)	Control eyes (*n* = 56)	*p* (with vs. without wedge)	*p* (glaucoma vs. control eyes)
Superficial vascular complex	20.05 (6.47)	19.37 (6.09)	20.21 (6.56)	23.54 (5.90)	0.149	0.001*
Deep capillary plexus	19.66 (8.29)	19.80 (7.03)	19.62 (8.60)	20.43 (8.37)	0.746	0.579

*Note*: Vessel density at each level (superficial vascular complex and deep capillary plexus) for cohort two with and without vascular wedge defects, and controls. Data are mean (*SD*). *p*‐values calculated by generalised estimating equations model.

To investigate the impact of glaucoma severity, as determined by VF examination, cohort two were stratified by mean deviation (MD) into three groups based on the distribution of MD within the cohort: least severe MD ≥ −2.00 (actual MD of +2.38 to −1.97, mean = −0.28, *n* = 146), moderately severe with MD between −2.01 and − 6.00 (actual MD of −2.01 to −5.81, mean = −3.32, *n* = 50) and most severe MD ≤ −6.01 (actual MD of −6.60 to −19.24, mean = −10.18, *n* = 12). SVC and DCP global macular densities were similar across all VF severity groups (Table 5). Vascular defects were present in 16 least severe (11%), 19 moderately severe (38%) and six (50%) most severe eyes, which was significantly different between groups (*p* < 0.001).

**TABLE 5:** Global macular vessel density at the superficial and deep vascular levels by disease severity

Vessel density (%)	Least severe (a) (*n* = 146 eyes)	Moderately severe (b) (*n* = 50 eyes)	Most severe (c) (*n* = 12 eyes)	*p* (a and b)	*p* (a and c)	*p* (b and c)
Superficial vascular complex	20.34 (6.80)	19.66 (5.43)	18.29 (6.53)	0.165	0.310	0.179
Deep capillary plexus	19.00 (8.73)	21.52 (6.92)	19.78 (7.50)	0.132	0.004*	0.197

*Note*: Vessel density at each level (superficial vascular complex and deep capillary plexus) by disease severity as determined by VF exam. Data are mean (*SD*). *p*‐values calculated by generalised estimating equation model.

VF defects were scrutinised in eyes that presented with OCTA vascular wedges in both cohort one and cohort two to determine the pattern of presentation of VF loss. VF defect distribution was as follows: 28 eyes had no defined VF loss, 7 eyes had a superior nasal step, 1 eye had an inferior nasal step, 1 eye had both superior and inferior nasal steps, 15 eyes had a superior arcuate, 11 eyes had an inferior arcuate, 3 eyes had superior hemispheric loss, 7 eyes had a superior paracentral defect, 1 eye had both inferior arcuate and superior paracentral loss, 1 eye had an inferior paracentral defect and 7 eyes had a nonspecific loss. VF scans for four eyes were unreliable and excluded. When VF defects and vascular wedges were both present, they appeared in opposing (corresponding) hemispheres. In eyes with both inferior and superior vascular wedge defects, bihemispheric VF loss was visible.

Refractive error, expressed as spherical equivalent, was analysed as a measure of myopia; high myopes were not excluded. Within cohort two, spherical equivalent averaged −0.06D ± 1.96 (range of −6.37 to 4.75D). There was no difference in spherical equivalent between those with vascular wedge defects and those without (*p* = 0.195).

Regarding RNFL and GCLIPL thinning, those with vascular wedge defects compared with those without had, on average, 8 μm thinner RNFL and 7 μm thinner GCLIPL (Table 6). Both RNFL and GCLIPL were thickest in control eyes. OCT scan quality ranged from 21 to 41 with mean qualities of RNFL = 29.78 ± 3.17 and GCLIPL = 32.37 ± 3.64.

**TABLE 6:** Retinal nerve fibre layer and ganglion cell layer inner plexiform layer thicknesses in eyes with and without vascular wedge defects and controls

Retinal layer thickness (μm)	Cohort two (*n* = 208 eyes)	With wedge defects (*n* = 41 eyes)	Without wedge defects (*n* = 167 eyes)	Control eyes (*n* = 56)	*p* (with vs. without wedge	*p* (glaucoma vs. control eyes)
Retinal nerve fibre layer	79.62 (13.69)	72.70 (12.12)	81.32 (13.55)	95.75 (9.75)	0.001*	<0.001*
Ganglion cell layer inner plexiform layer	81.27 (11.11)	75.35 (11.44)	82.61 (10.62)	90.09 (8.25)	0.003*	<0.001*

*Note*: Thickness of the retinal nerve fibre layer and ganglion cell layer inner plexiform layer as determined by OCT exam in cohort two, with and without vascular wedge defects and controls. Data are mean (*SD*). *p*‐values calculated by generalised estimating equation model.

**FIGURE 3:**
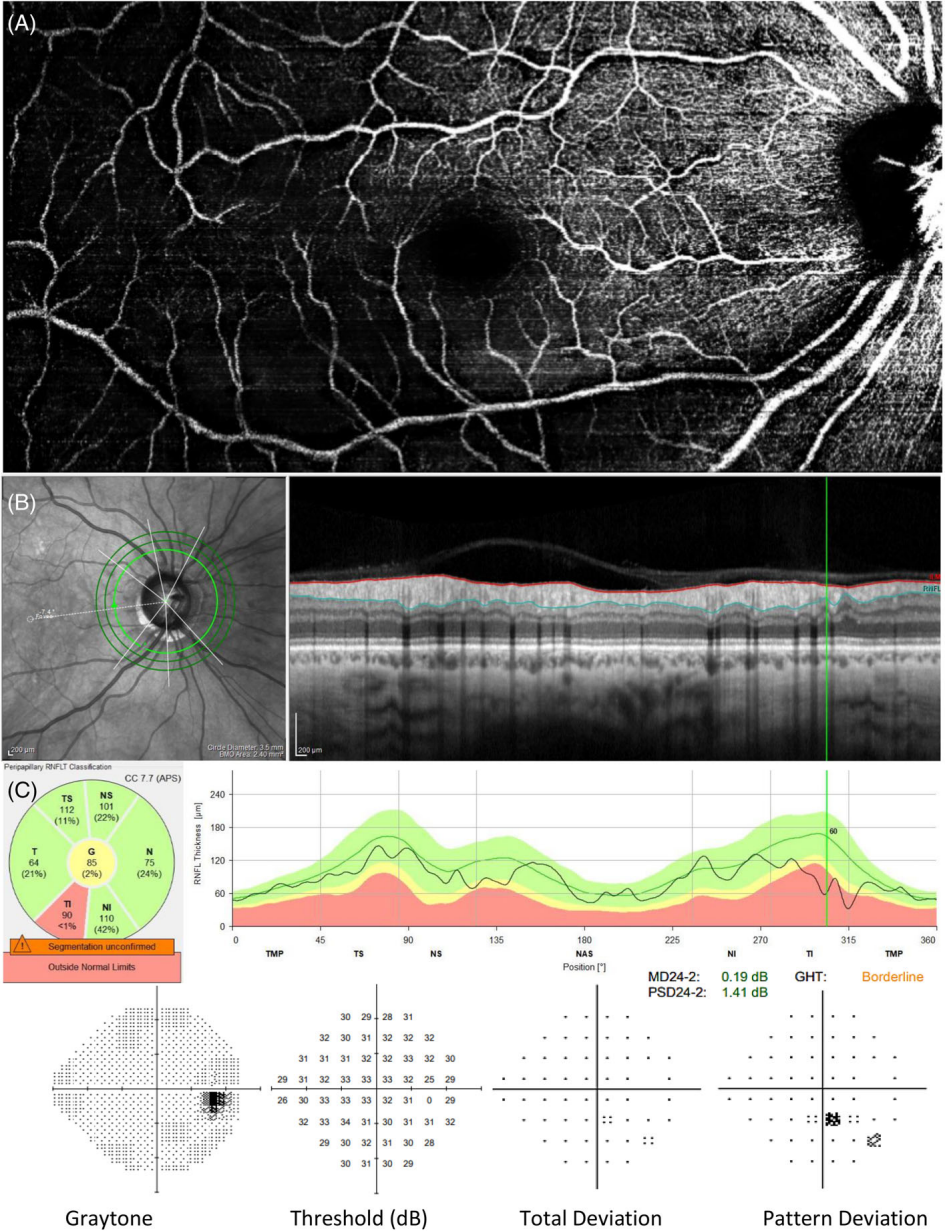
Example of vascular wedge defect presentation in a patient with preperimetric glaucoma. The eye shows a pronounced inferotemporal vascular wedge defect (A) visible on the Spectralis, Heidelberg OCTA macula scan. There is a small yet significant RNFL wedge in the same region as shown on the Spectralis, Heidelberg RNFL thickness output and classification chart (B). This individual did not show signs of functional loss at this visit, as seen on the Humphrey 24–2 VF examination (C)

RNFL sectors were assessed for significant and borderline thickness loss. In cohort two eyes, there were 189 sectors flagged as significant and an additional 191 flagged as borderline, on the Spectralis RNFLT classification chart. Of which, the majority were located in either the inferotemporal (72 significant, 31 borderline) or superotemporal (38 significant, 25 borderline) region (data unavailable for two eyes), as expected for glaucomatous pathology. Vascular wedge defects were always associated with RNFL loss in the same region (e.g., in Figure 3), with 37 eyes classified (by the system) as showing significant loss, and 4 eyes classified as borderline. In those with vascular wedges compared with those without, RNFL sectorial analysis revealed significantly thinner temporal inferior, temporal, and temporal superior regions (all *p* ≤ 0.001); no differences in nasal inferior, nasal or nasal superior (*p* = 0.613, *p* = 0.955 and *p* = 0.886, respectively). Similarly, GCLIPL was significantly thinner in the temporal inferior, inferior, and temporal superior sectors (*p* < 0.001, *p* < 0.001, and *p* = 0.007, respectively); no differences in nasal inferior, nasal superior, or superior (*p* = 0.097, *p* = 0.693 and *p* = 0.683, respectively).

## 4 DISCUSSION

This study investigated the prevalence and characteristics of vascular wedge defects in POAG. From the prevalence study (cohort one), we found vascular wedges present in 13% of eyes including glaucoma suspects, preperimetric, early glaucoma cases and ocular hypertensives. The in‐depth investigation (cohort two, which included more advanced cases) revealed vascular wedges in 19.7% of eyes, sometimes prior to major structural or functional loss. When the results from each cohort were combined, vascular wedge defect prevalence was around 16%.

Vascular wedges had strong concordance with RNFL defects and, similar to previous RNFL studies, were most often located inferiorly.^19^ While the association between vascular loss and RNFL loss is well established,^7^, ^8^, ^9^, ^20^ there is limited research which explores this relationship and vascular wedge defects in‐depth. RNFL wedge defects have long been a recognised glaucoma feature,^5^, ^21^ strongly associated with disease progression and disc haemorrhages.^5^

History of DH was determined to be more likely in eyes with vascular wedge defects than in eyes without. Similarly, LeTran observed a strong relationship between DH history and vascular wedge defects, with 65% of POAG eyes with both vascular wedges and DH having hemispheric concordance of these events.^11^ Lee et al. observed increased choroidal vessel density loss in localised regions which spatially corresponded to DH location in POAG eyes.^22^ Further, Nishida et al. observed in a longitudinal study that DH presence was a predictor of faster vessel density loss.^23^ DH presence has long been established as being associated with glaucoma progression, despite this, the underlying link in pathology is still in debate.^24^ DH cause is often hypothesised to be vascular in origin, due to microinfarctions or small branch vein occlusions, or due to structural disc change with disruption of supporting lamina connective tissue.^25^, ^26^ The relationship between DH and localised vessel density loss suggests that better understanding the cause of DH may help explain the origins of vascular wedge defects. Future studies could further investigate this relationship and the relative timing and location of their appearance.

The association between systemic vascular conditions, in particular hypertension, and glaucoma has previously been reported.^2^, ^27^, ^28^ Specifically, RNFL defects have been associated with vascular disease, in particular multiple RNFL defects has been linked to hypertension, end‐stage renal disease and cerebrovascular disease.^29^, ^30^ Various systemic vascular‐related conditions, including diabetes and hypertension have also been associated with low tension glaucoma.^31^, ^32^ We explored the notion that vascular wedge defects may be attributable to systemic vascular disease (self‐reported hypertension and diabetes) however since no statistical relationship was found, we have no reason to suggest that they were not glaucomatous in origin.

Interestingly, retinal microvascular changes have been shown to precede the onset of severe hypertension in a large older population study.^33^ It would therefore be informative to follow the current study cohorts to determine if localised vascular defects are associated with future development of systemic vascular conditions including hypertension.

In cohort one, eyes with vascular wedge defects were associated with significantly lower IOP than eyes without wedge defects. Initially this may seem to suggest that vascular wedge defects are more likely to be present in eyes with normal tension glaucoma compared with other types of POAG, similar to RNFL wedges.^5^ However, comparing eyes with and without vascular wedges in cohort two suggests that IOP was not significantly different. The more likely explanation for the discrepancy in cohort one is the high number of ocular hypertensive individuals without vascular wedge defects (*n* = 44) compared with those with vascular wedges (*n* = 1). Future large cohort studies should subdivide the cohort by IOP, and document IOP prior to treatment and at multiple timepoints to determine if it is a factor involved in the incidence or progression of vascular wedge defects.

Global macular vessel density was not significantly different between those with and without vascular wedge defects, yet cohort two had significantly lower SVC density than controls. Vessel density was also similar across severity groups (as outlined in Table 5) with a significant difference only found in the DCP between least and most severe eyes. However, considering the reduced reliability of the DCP scan quality, this warrants further investigation. The similarity in global macular vessel density values may be explained by the high number of preperimetric glaucoma eyes (98, 47%). Preperimetric vessel density has previously been reported as similar to normal‐tension glaucoma eyes and healthy controls,^8^, ^34^ and statistically different from early manifest glaucoma and controls.^35^ Peripapillary capillary density has been shown to be progressively lower with increasing glaucoma severity.^36^ Previous studies often analyse the 3 × 3 mm scan which has a greater resolution than the 6 × 6 mm (cohort one) and 8.7 × 4.4 mm (cohort two) scans however, larger scan area was chosen to best visualise vascular wedge projection, despite the increase in capillary network detail from smaller scans which may influence overall density results. In support of wider field analysis, Akiyama et al. reported that in early glaucoma eyes, a perifoveal annulus around the foveal avascular zone (with outer limits of 6 mm and inner of 3 mm) had a stronger structure–function relationship with VF than a smaller parafoveal annulus (outer limits of 3 mm, inner of 1 mm).^37^ The current study's scan size could still be considered a limitation in that wider‐field images were not obtained, which may have increased wedge identification, and the ability to quantify a larger proportion of wedge defects, however, this would have further reduced resolution and observable detail. Therefore, scan size and the analysis rigour must be noted when comparing results across studies.

VF analysis revealed that when both field loss and vascular wedge were present, they occurred in opposite hemispheres. Similarly, Ichiyama et al. observed concordant VF loss with localised reduced vessel density.^7^ LeTran et al. specified paracentral defects as being associated with vascular wedge defects, however, it is unclear whether other patterns of field loss were not reported or not present.^11^ The current study includes a high proportion of preperimetric and early‐stage POAG eyes with only minor VF loss. In eyes with vascular wedge defects, we found most showed no definite VF loss (34%), however, when VF loss had occurred, it was most commonly arcuate in presentation, either superior (18%) or inferior (15%). The high number of preperimetric and early‐stage POAG as well as the cross‐sectional study design limited the functional analysis that could be performed, therefore limiting the ability to draw conclusion of the correlation of vascular wedge defects with specific patterns of VF loss.

In a longitudinal study, Kamalipour et al. measured VF progression for a minimum of 3 years before imaging the macula of POAG eyes with OCTA and found that VF progression was associated with subsequent reduced vessel density of the superficial level.^38^ Further research could extend the current study to determine whether localised vessel density affects this relationship and the relative location of functional loss.

Cohorts one and two were examined using different OCTA systems‐Angioplex, Zeiss and Spectralis, Heidelberg, which is considered a study limitation. This was because participants were recruited from a multicentre study and analysed in different locations with different system access. To best account for system differences, cohorts were separated, thereby no statistical analysis included data from both systems. Our previous research revealed that Zeiss and Heidelberg OCT system data are not comparable at a single visit, yet can be used to compare rates of change in glaucoma.^39^ Further, Spectralis SVC and DCP layers were chosen to best match Angioplex bounds, and vessel density was measured externally (ImageJ). However, despite these attempts the use of two systems may still have an impact, for example, wedge defects may be more detectable with one device. Our impression is that defect detection is similar using both systems, but direct comparison cannot be done since no subjects were tested using both systems.

There was a discrepancy in the relationship between corneal thickness and vascular wedge defect presence, being that in cohort one, eyes with wedge defects had lower corneal thickness, whereas in cohort two, eyes with wedge defects had non‐significantly thicker corneas compared with eyes without wedge defects. Reduced corneal thickness is typically associated with glaucoma progression,^40^ which supports the reduced thickness in the eyes with vascular wedges in cohort one which were more likely to have glaucoma progression. Yet, LeTran et al. observed higher corneal thickness in eyes with vascular wedge defects compared with those with non‐specific defects or profound loss,^11^ supporting the trend in cohort two. Future studies are needed with larger cohorts to determine the true relationship between wedge defects and corneal thickness.

As this was a cross‐sectional study, the temporal relationship between vascular and RNFL wedge defects or functional change could not be deduced. Considering all vascular wedge defects were associated with RNFL defects, yet not all RNFL defects corresponded with vascular defects, one could infer that vascular wedge defects arise later in disease progression; further supported by the proportion of vascular wedges in advanced eyes. Furthermore, vascular wedges do not anatomically coincide with diffuse capillary bed damage, rather they are localised to the bounds of nerve fibre bundles, suggesting secondary degeneration due to RNFL loss. A similar conclusion was reached by Lee et al. in normal‐tension glaucoma.^9^ However Moghimi et al. showed that lower macular vessel density at baseline was associated with subsequent faster RNFL progression.^14^ Functionally, Wang et al. observed reduced peripapillary vessel density in the superotemporal region to be associated with subsequent VF progression in a normal‐tension glaucoma cohort, suggesting vascular dysfunction precedes functional change.^41^ It is possible that glaucoma phenotypes may present in a heterogenous manner, and since RNFL wedges were at times less pronounced than their vascular counterpart, the temporal relationship can only be determined by follow‐up studies.

### 4.1 Conclusion

This study reports on the location and prevalence of vascular wedge defects in POAG, and their relationship to glaucoma parameters. Vascular wedge defects were found in ~16% of all glaucoma cases and were present even in the early stages. Vascular wedges were strongly associated with focal RNFL loss in the same region and could not be explained by systemic vascular conditions. Further research is needed to explore these defects, in particular their temporal relationship with clinical glaucoma parameters to determine if the vascular dropout is primary or secondary.

## CONFLICT OF INTEREST

The authors declare no conflicts of interest.
